# Urine metabolomic responses to aerobic and resistance training in rats under chronic unpredictable mild stress

**DOI:** 10.1371/journal.pone.0237377

**Published:** 2020-08-12

**Authors:** Yumei Han, Yi Jia, Junsheng Tian, Shi Zhou, Anping Chen, Xin Luo

**Affiliations:** 1 School of Physical Education, Shanxi University, Taiyuan, Shanxi Province, China; 2 Modern Research Center for Traditional Chinese Medicine, Shanxi University, Taiyuan, Shanxi Province, China; 3 School of Health and Human Sciences, Southern Cross University, Lismore, New South Wales, Australia; University of New Brunswick, CANADA

## Abstract

**Background:**

It is known that bioenergetics of aerobic and resistance exercise are not the same but both can effectively improve depression. However, it is not clear whether and how different types of exercise can influence depression through the same metabolic regulatory system. Metabolomics provides a way to study the correlation between metabolites and changes in exercise and/or diseases through the quantitative analysis of all metabolites in the organism. The objective of this study was to investigate the effects of aerobic and resistance training on urinary metabolites by metabolomics analysis in a rodent model of depression.

**Methods:**

Male Sprague-Dawley rats were given chronic unpredictable mild stress (CUMS) for eight weeks. The validity of the modeling was assessed by behavioral indices. After four weeks of CUMS, the rats that developed depression were randomly divided into a depression control group, an aerobic training group and a resistance training group. There was also a normal control group. From week 5, the rats in the exercise groups were trained for 30 min per day, five days per week, for four weeks. The urine samples were collected pre and post the training program, and analyzed by proton nuclear magnetic resonance (^1^H-NMR) spectroscopy.

**Results:**

Both types of training improved depression-like behavior in CUMS rats. Compared with normal control, 21 potential biomarkers were identified in the urine of CUMS rats, mainly involved in energy, amino acids and intestinal microbial metabolic pathways. Common responses to the training were found in the two exercise groups that the levels of glutamine, acetone and creatine were significantly recalled (all P<0.05) Aerobic training also resulted in changes in pyruvate and trigonelline, while resistance training modified α-Oxoglutarate, citric acid, and trimethylamine oxide (all P<0.05).

**Conclusions:**

Aerobic and resistance training resulted in common effects on the metabolic pathways of alanine-aspartate-glutamate, TCA cycle, and butyric acid. Aerobic training also had effects on glycolysis or gluconeogenesis and pyruvate metabolism, while resistance training had additional effect on intestinal microbial metabolism.

## Introduction

Depression is a common neuropsychiatric disorder. The World Health Organization (WHO) announced in March 2018 that more than 300 million people worldwide suffer from depression. Worst of all, nearly 800,000 people suicide each year that are associated with depression [1]. WHO predicts that, by 2020 depression will become the second largest disabling disease in the world [2]. The current treatment for depression is mainly by medication, such as tricyclic antidepressants, monoamine oxidase inhibitors and selective monoamine reuptake inhibitors, etc. [3]. Although the medication can ameliorate depressive symptoms, many patients are unable to continue with the treatment because of the side effects, such as sexual dysfunction, gastrointestinal reactions, anxiety symptoms, palpitations, and sleepiness [4, 5]. Therefore, it is important to develop new, alternative or complementary therapies for depression. Animal models have been employed as an important tool for studying the pathogenesis of depression. Among them, the rat model with chronic unpredictable mild stress (CUMS) can mimic many of the core symptoms of human depression and is the most effective and commonly used depression model [6].

It is known that exercise interventions can improve depression [7–9]. For example, there have been reports that exercise can improve neurobiological function, neuroimmune function and brain mitochondrial function in depression-like rats [10–14]. Compared with medication for depression, exercise therapy has advantages such as controllable exercise intensities, simplicity in implementation, no toxic side effects, and relatively low cost [15]. There has been a rapid development in the field of exercise therapy for depression in recent years, with broad application prospects [16]. There is a variety types of exercise that can be used in therapy; among them aerobic and resistance exercises are commonly prescribed. Both aerobic [17, 18] and resistance [19, 20] exercise interventions have shown antidepressant effects, however their underlying mechanisms have not been fully elucidated. Previous literature has focused more on the effects of aerobic exercise on depression at the molecular biological level [17, 18]. This may be due to the facts that application of resistance exercise intervention in depression models requires a higher level of skills, specific facilities and continuous supervision in practice. These factors may be potential obstacles that make resistance training programs more difficult to implement than aerobic training programs. It is well known that aerobic exercise relies on mainly the aerobic energy system to produce ATP. In resistance exercise, high intensity muscle contractions rely mainly on the immediate (anaerobic) energy sources that are replenished by the aerobic metabolism during the recovery intervals [21]. Therefore, different types of exercise, in respect of exercise intensity, duration and energy sources, may result in different impacts when being used as interventions for health conditions. However, an interesting question is that, from the viewpoint of metabolism, why and how aerobic and resistance exercise interventions can result in similar antidepressant effects.

Metabolomics is a relatively new omics method that follows genomics, transcriptomics and proteomics and provides a wealth of information about metabolic disorders in human diseases [22]. The alternative term metabonomics was originally defined as "the study of metabolic responses to disease, environmental change, or genetic modification in organisms", and metabolomics was later defined as "the comprehensive analysis by which all metabolites in a biological system are identified and quantified" [23]. The two terms are now used interchangeably, but in this article we will refer to the latter throughout. Since most metabolites are produced by enzymatic reactions through expression of genes, metabolomics can demonstrate the association between the genotypes and phenotypes, and allows a more direct measurement of pathophysiological changes than other omics such as genomics and proteomics [24]. After nearly 20 years of development, metabolomics has made significant breakthroughs, especially in the studies of phenotypes, toxicology, disease-related biomarkers and molecular mechanisms [25]. Various analytical techniques such as nuclear magnetic resonance (NMR), gas chromatography-mass spectrometry (GC-MS), and liquid chromatography-mass spectrometry (LC-MS) have been widely used in metabolomics research. Compared with GC-MS and LC-MS methods, NMR is preferred by many researchers because of its inherent advantages such as ease of quantification, non-destructiveness to samples, and unbiased metabolites [26, 27].

This study was the first to use the NMR metabolomics method to examine and compare the antidepressant effects of aerobic and resistance exercise interventions on the changes of metabolites in urine and behavioral indicators in a rodent model of depression. The aims of the study were (1) to identify potential biomarkers in metabolic pathways that were associated with the development of depression, and (2) to explore and compare the potential mechanisms underlying the effects of the aerobic and resistance exercise interventions in improving depression symptoms through the pathway analysis. We hypothesized that depression is associated with metabolic disorders in the body; and both aerobic and resistance training can improve depression by regulating certain common or different metabolic pathways in energy metabolism.

## Materials and methods

### Subjects

Male Sprague-Dawley rats, SPF grade, eight weeks old with body mass of 180~200 g, were obtained from Beijing Weitong Lihua Experimental Animal Technology Co., LTD (Animal License Number: SCXK, Beijing, 2016–0006). The animals were housed in an air-conditioned room with ambient temperature 23±1.5 (standard deviation) °C, relative humidity 45±15%, light on/off for 12/12 h.

### Ethical approval

This study was approved by the Committee of Scientific Research of Shanxi University (Approval Number: SXULL2018006). Maximal effort was made to minimize animal suffering and the number of animals necessary for the acquisition of reliable data.

### Chronic unpredictable mild stress (CUMS) modeling and grouping

The subjects’ body weight, sucrose preference rate, and the number of crossing in an open field test were used as indicators of depression [28], and measured after one-week adaptation to the laboratory environment before commencement of the experiment. The rats were first randomly divided into a normal control group (C), and three experimental groups: CUMS control (D), aerobic training (A) and resistance training (R). Except group C, the rats in other groups were single caged (cage size: length 420 mm, width 240 mm and height 240 mm). The modeling procedure followed that of Willner [29] with minor modifications. The experimental groups received unpredictable stimulations in a random order, with one type of stimulus was given in each day and different stimuli were given in consecutive days of each week. The following modeling stimuli were applied: no water for 24 h, fasting for 24 h, 4°C ice bath for 5 min, ultrasonic stimulation for 3 h, thermal stimulation for 10 min, and electric shocks to feet (voltage 36 V, shock interval 10 s, for 10 times), tail clipping for 2 min, movement restraint for 3 h, day and night reversal (placed under fluorescent light for 24 h).

After four weeks of modeling, the rats were tested (described below) for depression-like syndromes. Only stress-susceptible rats from the three modeling groups were selected for subsequent interventions. The rats in groups A and R were given the corresponding treatments for further four weeks, while continuously receiving the modeling stimuli together with group D.

### Evaluation of model validity

The validity of the modeling was evaluated by the changes in body weight, sucrose preference test and the number of crossing and standing in an open field test, on the last day of each week for all groups.

#### Body weight measurement (BW)

The BW was measured between 3 pm and 5 pm on the last day of each week.

#### Sucrose preference test (SPT)

The SPT is a common method to evaluate a core symptom of depression—anhedonia [30]. The SPT method used in this study was slightly modified from that of Yang et al. [31]. Each rat was given two bottles of 1% w/v sucrose solution (1 gram in 100 mL) that were placed above the cage for 24 hours. Then, one bottle was replaced with tap water for the next 24 hours. The rats were then deprived of water and food for 12 hours, then subjected to a sucrose preference text by given two pre-weighed solution vials: 100 ml 1% sucrose solution, and 100 mL tap water. The two vials were placed at the same height. The sucrose preference rate was determined by the consumption of the 1% sucrose solution relative to the total liquid intake, measured after three hours; i.e., sucrose preference rate (%) = sucrose intake (g) / total liquid intake (g) × 100%.

#### Open field test (OFT)

The OFT is commonly used to evaluate anxiety-like behavior and the effects of antidepressant treatment in rats [32]. The test was carried out as described by Gao et al. [33] with minor modifications. The periphery and bottom of the test space were made of black opaque metal sheets, with the dimension of length 100 cm, width 100 cm and height 70 cm. The floor area was divided into 25 squares of the same size. During the test, each rat was placed in the center of the area, and was allowed to explore freely for 5 min. The numbers of crossing and standing were recorded during the last four minutes by three trained technicians who were blind to the experimental groups. A crossing referred to entering into a new square with four paws, reflecting its ability to exercise. The standing referred to that the forelimbs of the rat raised from the ground, reflecting their ability to explore. After each test, the open area was washed with 70% ethanol to avoid any olfactory cues.

## Exercise protocols

### Aerobic training

From the fifth week with CUMS, rats in group A exercised on a motor-driven treadmill for 30 min, five times per week for four weeks. The treadmill speed started from 3 m/min for 5 min, increased to 5 m/min for 5 min, then to 8 m/min for 20 min. To diminish the stress from unfamiliar activities, rats were familiarized with the treadmill for three consecutive days (15 min per day) before the commencement of formal exercise intervention. They ran at the speed of 3 m/min for the first 5 min, 5 m/min for the second 5 min and 8 m/min for the last 5 min [19]. The slope of the treadmill belt was kept flat. During the exercise, rats in group C and group D were also placed in the same room, for a control of environmental factors. After the exercise, the treadmill belt was wiped with 70% ethanol solution and dried naturally to avoid any olfactory cues.

### Resistance training

**Fig 1:**
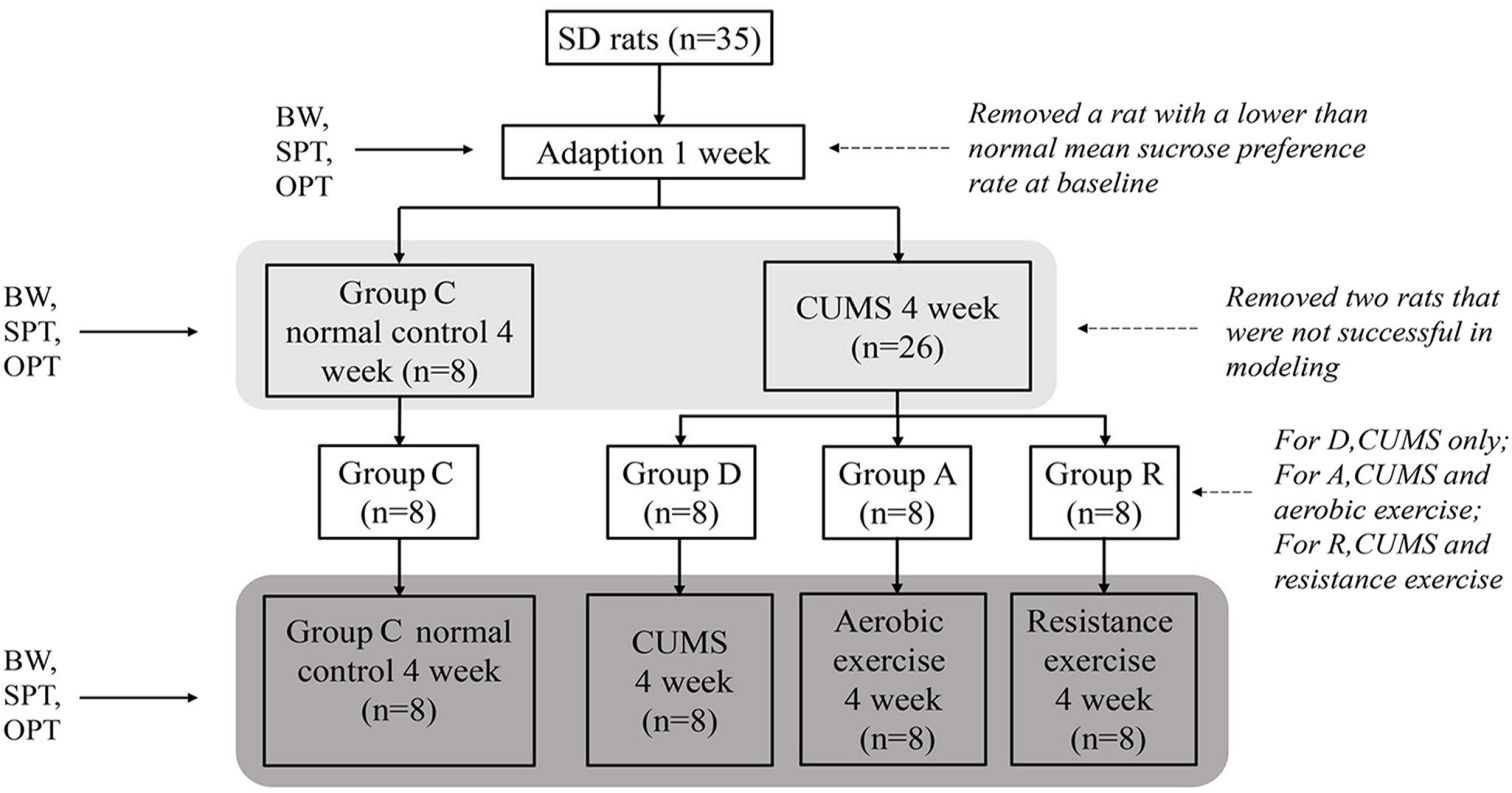
CUMS program and behavior test schedule. BW, body weight; SPT, sucrose preference test; OFT, open field test.

The resistance training program was set based on pilot trials, according to the literature [34, 35]. The rats were given three days pilot trials, and were trained to climb a vertical ladder (length 1.1 m, width 0.18 m, with 2 cm grid and 80 ° inclination). The rats climbed the ladder four times on the first day, six times on the next day, and eight times on day 3, with no additional load. Rats that could not climb the ladder were excluded from the study. After the third day, the eligible rats were trained by continuously climbed eight times in each session within 30 min, five sessions per week for four weeks, with incremental loads. The load was added to rat’s tail by taping (Vega tape) on pieces of lead chips according to the animal’s body weight. During the first training week the load was 25% of the baseline body weight, and 25% BW more was added in each following week till 100% BW in the fourth week. The animals that stopped climbing were touched on the back by a ruler or tweezers to encourage the movement. At the top of the ladder, there was a wooden box in which the rats could rest for two minutes before commencement of the next climbing repetition (Fig 1).

### Urine sample collection

Urine was collected on ice using metabolic cages at the end of the experiment from 9:00 pm to 9:00 am in the next day. The collected urine was centrifuged at 13,000 rpm for 20 min at 4°C to obtain the supernatant sample. The average sample size was 2 mL. The urine sample was stored at -80°C before analysis.

### NMR metabolomics

Urine sample pretreatment: Preparation of the samples for metabolomic analysis by ^1^H-NMR was performed as described previously [36]. 500 μL of urine sample was transferred to an Eppendorf tube after thawing, and 200 μL phosphate buffer solution (0.2 mol/L Na_2_HPO_4_, 0.2 mol/L NaH_2_PO_4_, pH = 7.40) containing D_2_O was added. Then an appropriate amount of 0.15% sodium 3-trimethylsilyl-(2,2,3,3-d4)-1-propionate (TSP) was added as a calibration of the zero point of chemical shift. The sample was then centrifuged at 13,000 rpm for 20 min at 4°C. 600 μL of the supernatant was placed in a nuclear magnetic tube with an inner diameter of 5 mm.

NMR detection conditions: The ^1^H-NMR spectra of the urine samples were obtained using a Bruker 600 MHz AVANCE III NMR spectrometer (Bruker Biospin, Rheinstetten, Germany) operating at a ^1^H frequency of 600.13 MHz and a temperature of 298 K. Samples were analyzed using the NOESY pulse sequence. The parameters were set to a spectral width of 8 kHz, a mixing time of 150 ms, a relaxation delay of 320 ms, sampling point of 64 k, and accumulation number of 64. The pre-saturation method was used to suppress the water peak during the relaxation delay, the spectrometer bias is set at the water peak position, and the free induction decay signal was converted into a ^1^H-NMR spectrum by Fourier transformation.

NMR spectrum processing: Urine ^1^H-NMR spectra were adjusted for baseline and phase distortions using the MestReNova software (ver. 8.0.1, Mestrelab Research, Santiago de Compostela, Spain). The chemical shift correction of the NMR spectra was performed based on the chemical shift (δ0.0) of TSP. With δ0.01 as the basic unit, the integral of the region of δ0.8~8.0 in the spectrum could be obtained, and the integral value corresponding to the chemical displacement value segment was acquired. In order to eliminate the influence of water peak and urea peak on the analysis results, the peak intensity of the δ4.6~6.2 interval was set to zero. Then, the normalization method was used to eliminate the influence of the difference in sample concentration, so that the data was limited to the range of δ0.0~1.0. After data normalization, multivariate statistical analysis was performed.

### Statistical analysis

All data were expressed as mean ± standard deviation (X ± SD, n = 8 per group). Analyses were performed using SPSS statistical package (IBM SPSS, ver. 19.0, Chicago, USA). The graphics were generated using Graph Pad Prism software (ver. 7.0, La Jolla, CA). Two-way ANOVA with repeated measures was used to analyze data from behavioral tests. If a significant main effect or interaction was detected, post-hoc test with Bonferroni adjustment was performed to identify where a significant difference existed. Differential metabolites were assessed by independent group t test (two tailed). Principal component analysis (PCA), partial least squares-discriminant analysis (PLS-DA) and orthogonal partial least squares discriminant analysis (OPLS-DA) were performed using SIMCA-P multivariate statistical analysis software (ver. 14.0, Umetrics, Umea, Sweden). The quality of the PLS-DA and OPLS-DA model was validated by the response values of the permutation test, in which the class membership was randomly shuffled 200 times.

## Results

### Behavioral changes

**Fig 2:**
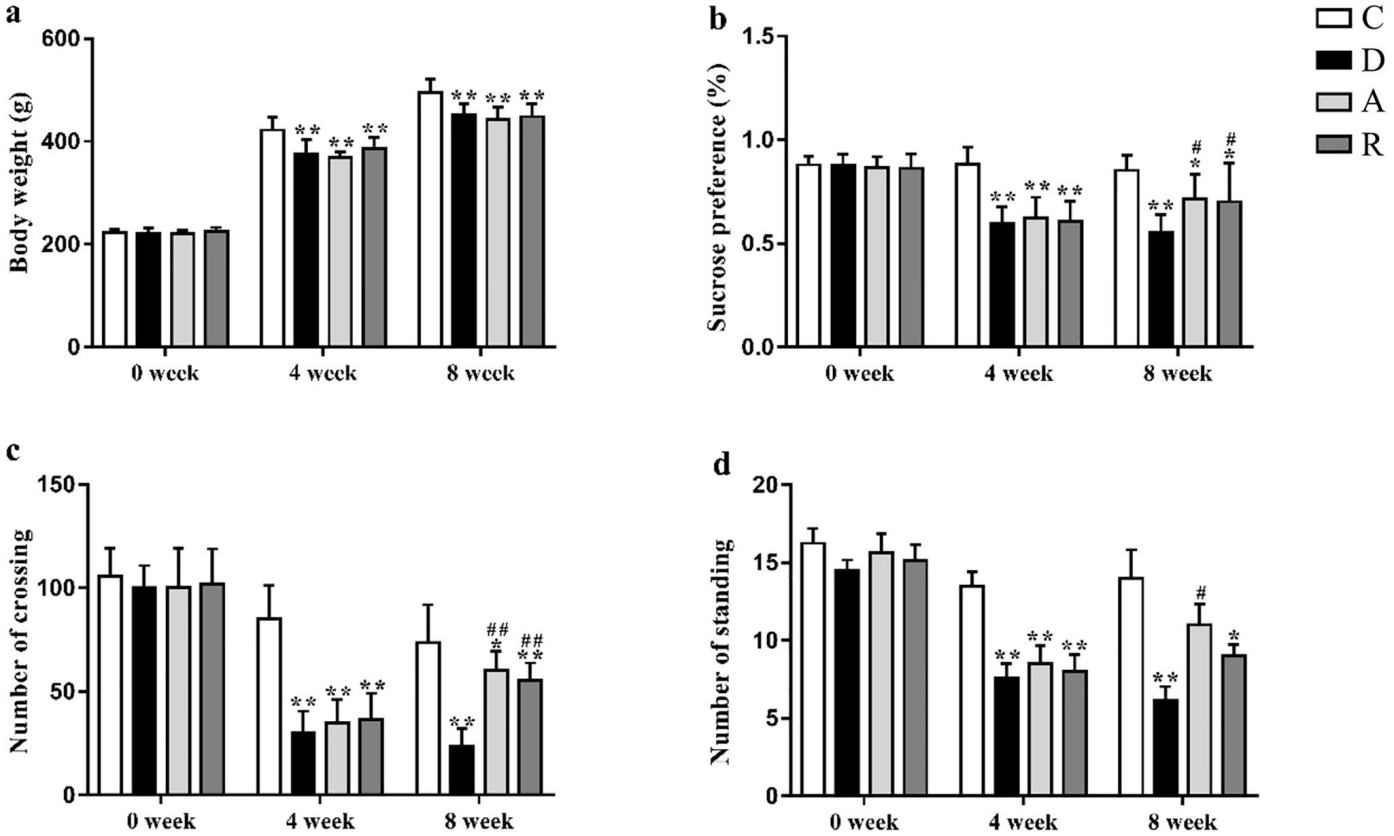
Effects of CUMS and different types of exercise training on depression-like behaviors. (a) Changes in body weight during the 8-week experiment; (b) sucrose preference rate; (c) number of crossing in open field test; and (d) number of standing in open field test. Data are presented as mean ± SD (n = 8 per group). *p < 0.05, **p < 0.01 versus normal control (Group C); ^#^p < 0.05, ^##^p < 0.01 versus CUMS control (Group D).

As shown in Fig 2, Two-way ANOVA analysis showed a significant effect of the intervention mode, and time, or a significant interaction between the two factors. After the four weeks of modeling, compared with group C, the BW, sucrose preference rate, and the number of crossing and standing were significantly lower (P<0.01), indicating that the CUMS depression-like model was established successfully. After the four weeks of exercise intervention period, the body weight, the sucrose preference rate and the number of crossings of the groups D, A and R was all significantly lower than that of the group C (P<0.05). At the same time, the number of standing in group D and group R was significantly lower as compared with group C (P<0.05). Compared with group D, the sucrose preference rate and the number of crossing in groups A and R were significantly increased, and the number of standing in group A was significantly increased (all P<0.05).

### Urine metabolomics

#### Urine NMR spectrum

**Fig 3:**
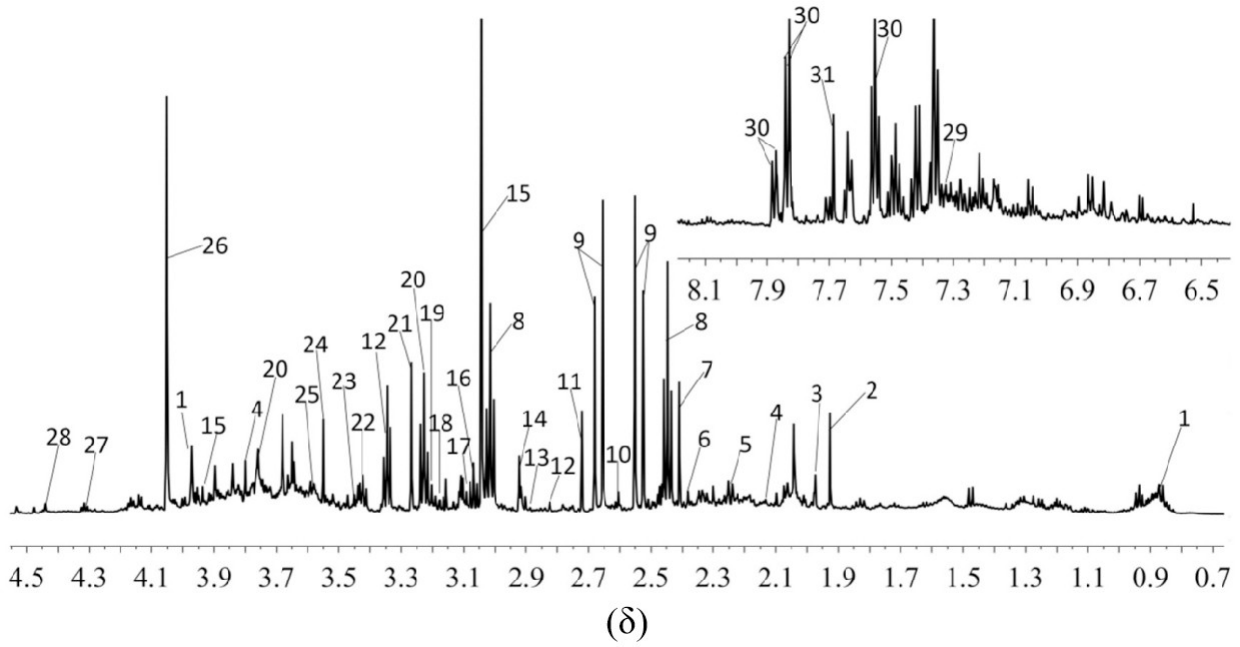
^1^H-NMR spectrum of the urine samples of the CUMS depression rats. (1) Pantothenate; (2) Acetate; (3) Glycoprotein; (4) Glutamine; (5) Acetone; (6) Pyruvate; (7) Succinate; (8) α-Oxoglutarate; (9) Citrate; (10) Methylamine; (11) Dimethylamine,DMA; (12) Methylguanidine; (13) Trimethylamine,TMA; (14) Dimethylglycine; (15) Creatine; (16) Putrescine; (17) Malonate; (18) Methylmalonate; (19) Glycerophosphocholine; (20) Arginine; (21) Trimethylamine N-oxide,TMAO; (22) Taurine; (23) Acetoacetate; (24) Glycine; (25) Threonine; (26) Creatinine; (27) Malate; (28) Trigonelline; (29) Phenylalanine; (30) Hippurate; and (31) Guanine.

A typical ^1^H-NMR spectrum of urine in CUMS depression rats is shown in Fig 3. Chenomx NMR Suite software (Chenomx Inc, Edmonton, AB, Canada, version 7.5) and HMDB database (http://www.hmdb.ca/) were used to identify the main chemical composition [36–38]. A total of 31 endogenous metabolites were identified, including tricarboxylic acid cycle intermediates, amino acids, and amines, etc. (Fig 3).

#### Multivariate statistical analysis

**Fig 4:**
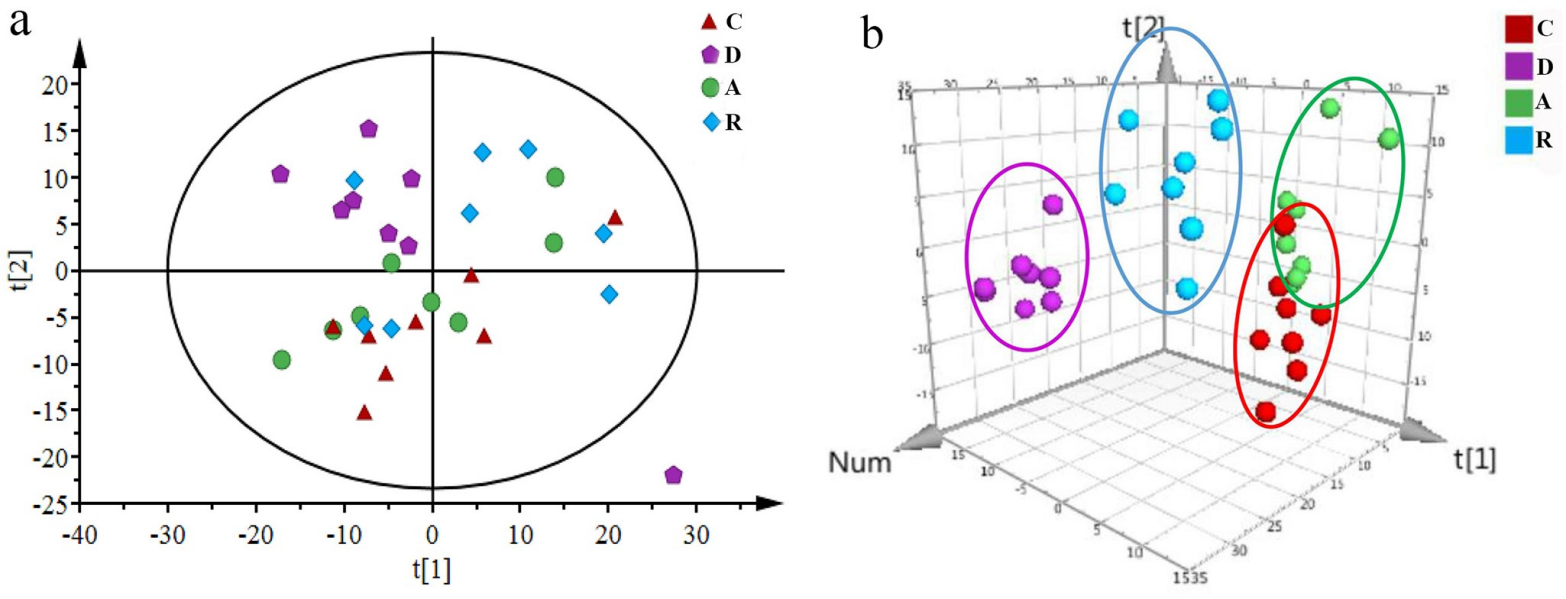
PCA (a) and 3D PLS-DA (b) scatter plot derived from ^1^H-NMR spectra of urine from rats in groups C, D, A and R.

Because the NMR spectrum analysis had provided more information, it was difficult to visually demonstrate the differences between the groups. Therefore, multivariate statistical methods were employed in further analysis. All samples were analyzed by PCA scatter plot to show the original classification status of the data (Fig 4a). It can be seen that the group C and the group D were clearly separated along t2, indicating that the depression model was successfully established. It can be seen from the 3D PLS-DA scatter gram (Fig 4b) that the samples of groups C, D, A and R can be clearly separated, and the samples of group A and group R are located between group C and group D, indicating that the aerobic and resistance training caused a certain level of improvement in rats with depression.

**Fig 5:**
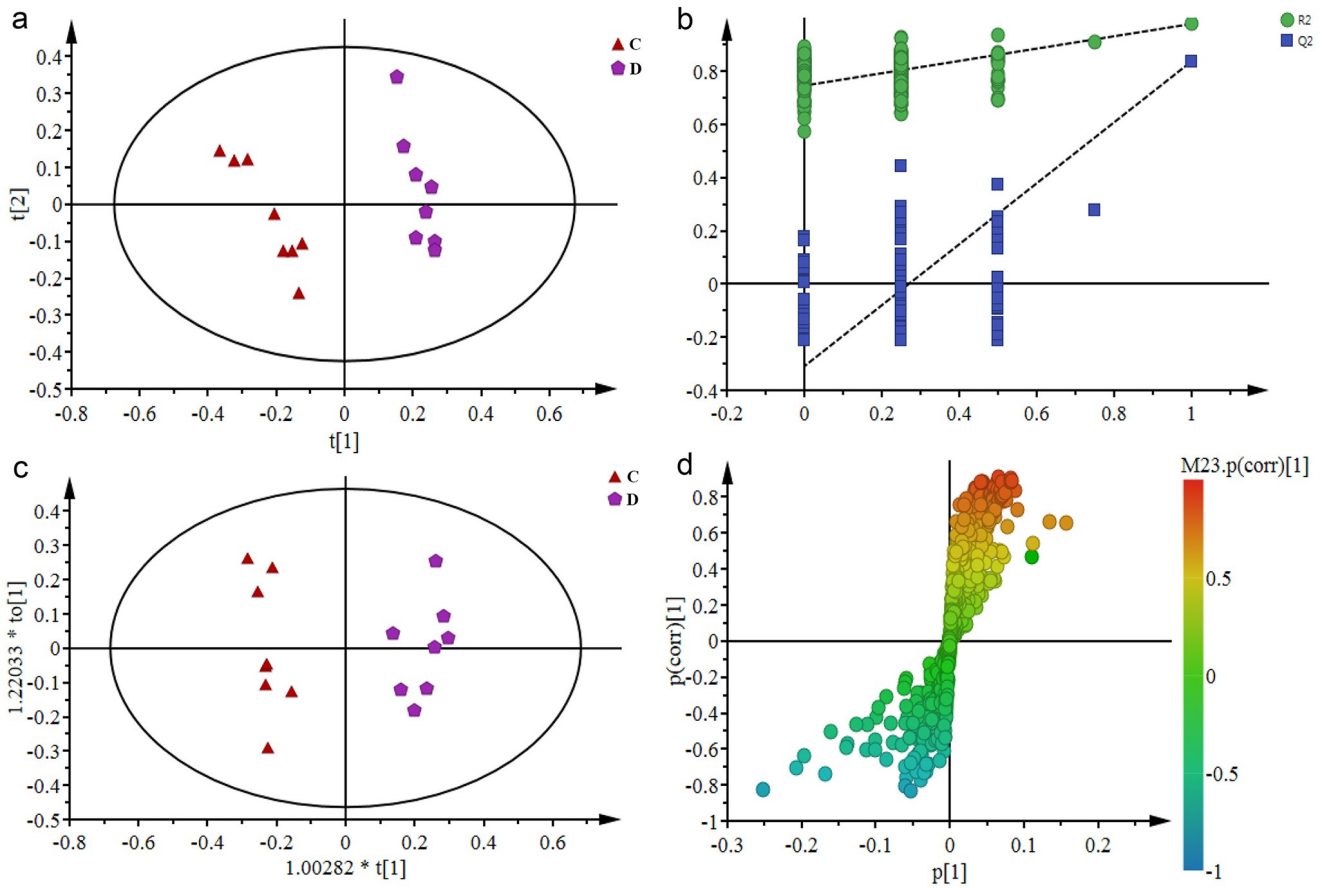
PCA scores plot (a), PLS-DA permutation test (b), OPLS-DA scores plot (c) and corresponding S-plot (d) in the urine samples of the normal control group (C) and the model control group (D).

To demonstrate the difference in endogenous metabolites in the urine of rats before and after modeling, the PCA scatter plot (Fig 5a) was first used to observe the trend of separation between the two control groups. It can be seen that the group C and the group D were clearly separated. As an unsupervised analysis method, PCA can reflect the original state of the data. However, during the experiment, the environment factors and some human errors could affect the experimental results. In order to filter out the noise signals that were not related to the model classification, the data were further processed using supervised PLS-DA analysis and OPLS-DA analysis for accurate results. The OPLS-DA analysis was based on the PLS-DA model. The permutation test results (Fig 5b) showed that all the simulated values of R^2^ and Q^2^ on the left side of the figure were smaller than the initial values (the green dot and the blue square in the upper right corner). Theoretically, the closer R^2^ and Q^2^ are to 1, the better the model fitting accuracy will be, and vice versa. In general, R^2^ and Q^2^ are better than 0.5. In this study, R^2^ = 0.963 and Q^2^ = 0.802 indicated that the modeling was effective and reliable. After the OPLS-DA analysis (Fig 5c), the S-plot (Fig 5d) was used to combine the VIP values (>1), and the independent sample t test was used to find the differential metabolites. A total of 21 potential biomarkers with significant differences (P<0.05) were obtained. The results (Table 1) showed that compared with the group C, the levels of acetate, glycoprotein, glutamine, dimethylamine, methylguanidine, trimethylamine, creatine, putrescine, trimethylamine oxide, acetoacetate, malate, trigonelline, and hippurate in group D increased, and the levels of acetone, pyruvate, α-Oxoglutarate, citrate, malonate, glycine, creatinine and guanine decreased.

**Table 1:** Relative peak area of 21 potential biomarkers in urine ^1^H-NMR spectra.

Potential biomarkers	Relative peak area(×1000)			
	Group C (n = 8)	Group D (n = 8)	Group A (n = 8)	Group R (n = 8)
Acetate	3.38±1.14	6.62±2.27**	7.92±5.83	10.10±5.52
Glycoprotein	1.21±0.12	1.66±0.40*	1.41±0.24	1.63±0.29
Glutamine	3.84±0.20	4.14±0.18**	3.78±0.18^##^^C^	3.83±0.34^#^
Acetone	3.47±0.30	2.96±0.15**	3.20±0.16^##^	3.20±0.16^##^
Pyruvate	2.02±0.17	1.67±0.10**	1.83±0.09^##^	1.78±0.19
α-Oxoglutarate	13.99±3.65	10.19±1.62*	11.91±2.83	11.94±1.63^#^
Citrate	14.43±2.72	10.28±1.38**	12.07±1.93	13.11±3.22^#^
Dimethylamine	2.54±0.97	3.63±0.87*	2.99±1.25	4.04±0.60
Methylguanidine	0.57±0.04	0.81±0.10**	0.74±0.12	0.83±0.16
Trimethylamine	0.66±0.08	0.89±0.17**	0.80±0.24	0.79±0.07
Creatine	2.07±0.13	2.71±0.24**	2.27±0.15^##^	2.42±0.24^#^
Putrescine	1.97±0.56	3.20±0.78**	3.57±1.50	3.91±1.48
Malonate	4.84±1.02	3.68±0.59*	4.41±1.24	4.31±1.01
TMAO	2.86±1.60	5.38±2.70*	3.46±1.35	3.05±0.49^#^
Acetoacetate	1.86±0.50	2.65±0.79*	2.82±0.93	2.80±0.50
Glycine	3.41±1.25	2.26±0.30*	2.76±1.20	2.98±0.19
Creatinine	25.14±2.51	18.73±4.84**	21.74±3.75	18.49±4.29
Malate	0.90±0.31	0.59±0.21*	0.69±0.24	0.72±0.46
Trigonelline	0.46±0.19	1.07±0.28**	0.75±0.26^#^	0.81±0.42
Hippurate	0.53±0.13	0.88±0.30*	0.60±0.28	1.17±0.64
Guanine	0.94±0.29	0.46±0.19**	0.60±0.29	0.33±0.17

^a^ All data were expressed as mean and standard deviation [SD].

^b^ Abbreviations: C = normal control group; D = CUMS control group; A = aerobic training group; R = resistance training group.

^c^ *p < 0.05, **p < 0.01 versus Group C; ^#^p < 0.05, ^##^p < 0.01 versus Group D.

**Fig 6:**
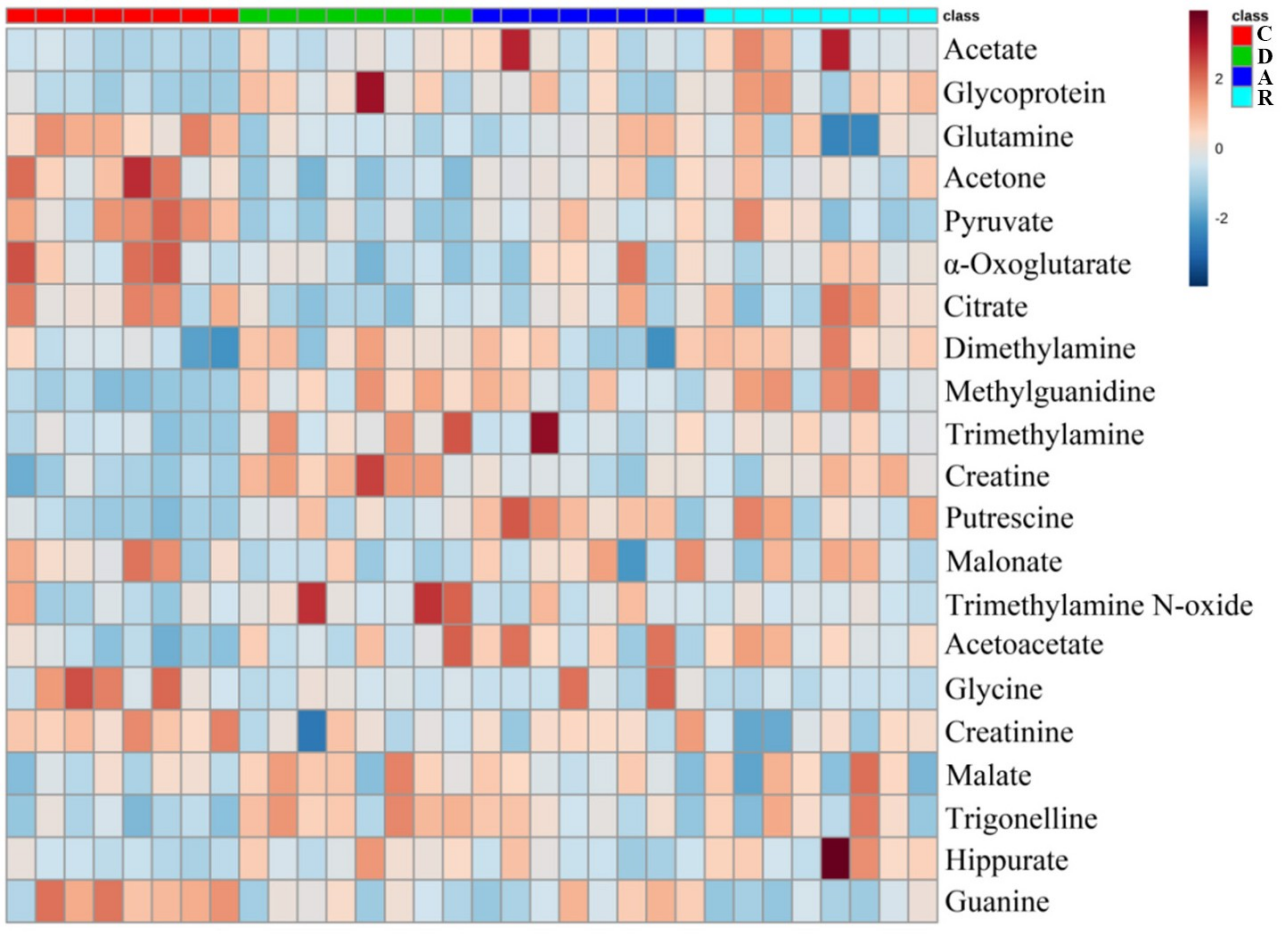
The heat map of potential biomarkers. The redder the color, the higher the expression level in urine. The bluer the color, the lower the expression level in urine.

There were five significant callbacks after the aerobic training, including glutamine, acetone, pyruvate, creatine, and trigonelline. There were six significant callbacks after the resistance training, including glutamine, acetone, α-Oxoglutarate, citrate, creatine, and trimethylamine oxide. The use of heat map analysis can more intuitively demonstrate the trend of 21 potential biomarkers in the urine samples of each group (Fig 6); the redder the color, the higher the concentration in the urine.

#### Pathway analysis

**Fig 7:**
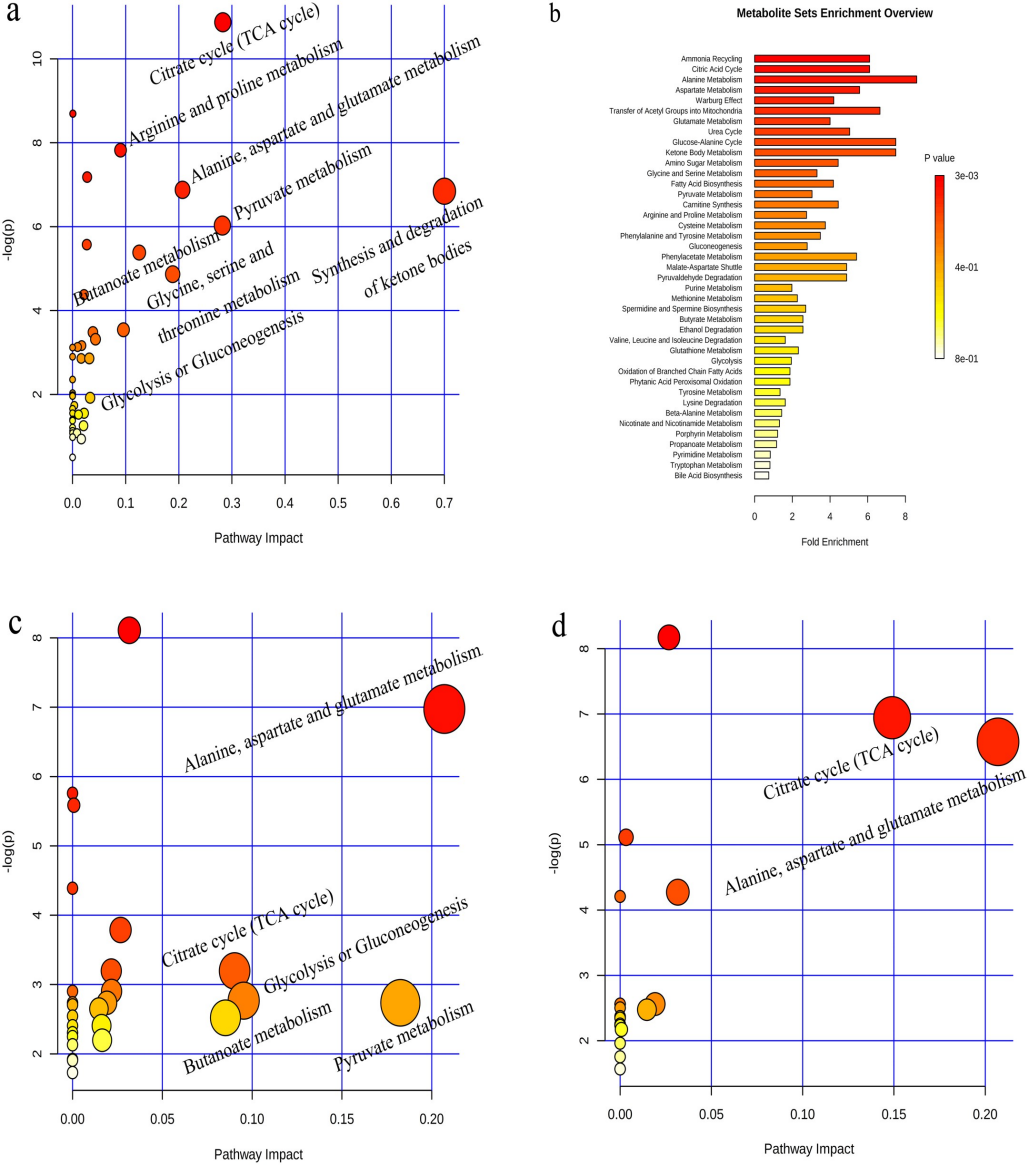
MetPA analysis of metabolic pathway (a. C vs D; c. D vs A; d. D vs R) and enrichment of metabolite sets (b).

**Fig 8:**
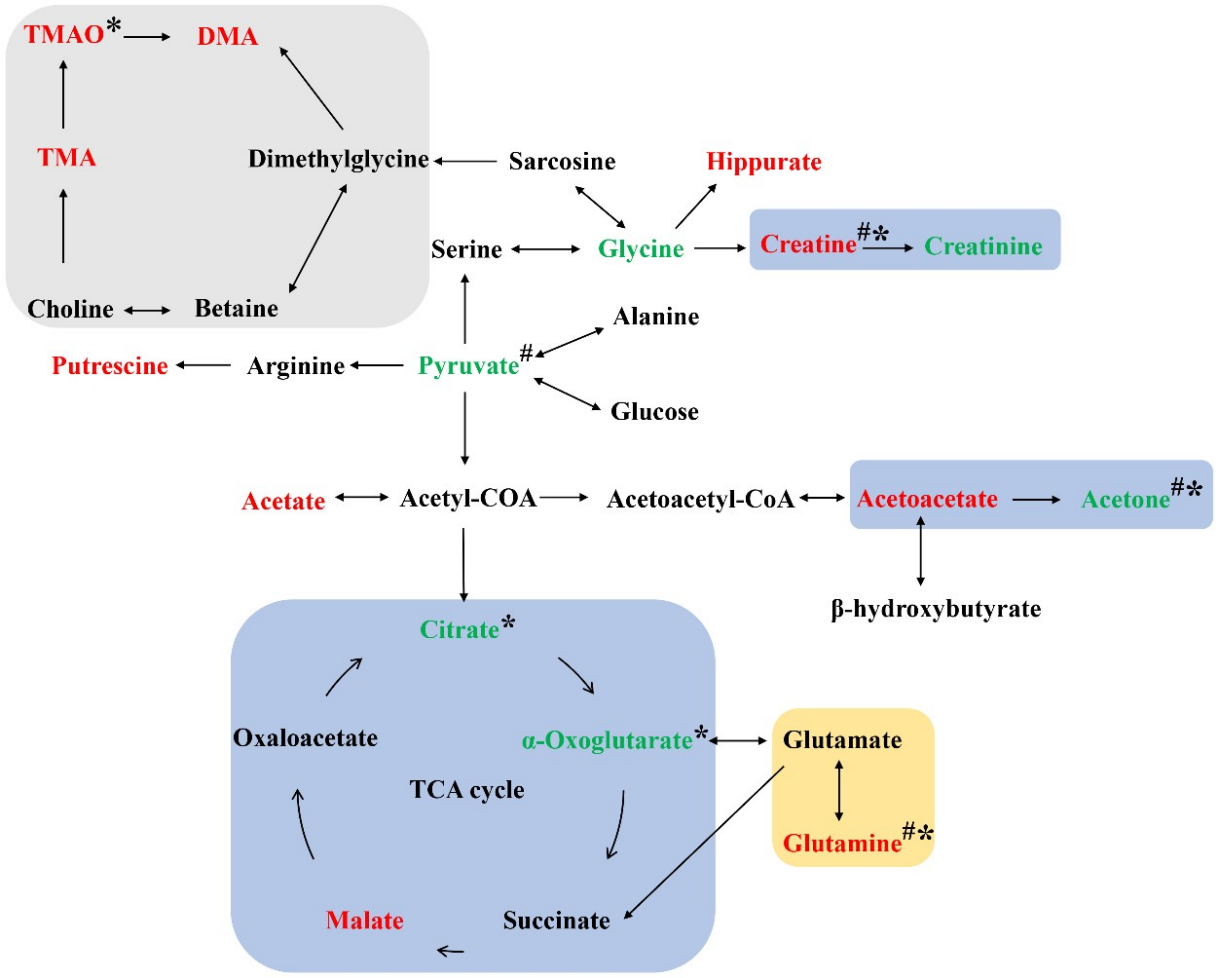
Metabolic pathway map of potential biomarkers in urine of CUMS rats. The red color labeled metabolites increased significantly and the green color labeled metabolites decreased significantly. Among them, ^#^ indicates that the change in a metabolite was significantly reversed by aerobic training, and * indicates that change in a metabolite was significantly reversed by resistance training.

To further determine the metabolic pathways affected by potential biomarkers in urine, all potential biomarkers were imported into the MetaboAnalyst 4.0 (http://www.metaboanalyst.ca/) online analysis system to obtain the pathway impact distribution maps and pathway enrichment map (Fig 7), and to explain the metabolic pathway changes caused by the modeling. In the enrichment map, the larger the enrichment factor, the more metabolites involved in this pathway; the larger the P value (the darker the color), the more obvious the changes in the metabolites or metabolic pathways involved [39]. In the pathway impact distribution map, the color and size of each circle were determined based on the P value and the channel influence factor. That is, the darker the color of the circle, the more metabolites involved in this pathway; and the larger the circle is, the more significant its role in the overall metabolic profile of the organism [40]. It can be seen from Fig 7a and 7b that CUMS modeling mainly affected alanine, aspartate and glutamate metabolism, tricarboxylic acid cycle (TCA cycle), glycolysis or gluconeogenesis, butyrate metabolism, pyruvate metabolism, arginine and proline metabolism, glycine, serine and threonine metabolism, and ketone body synthesis and degradation of these eight metabolic pathways. It can be seen from Fig 7c and 7d that the aerobic training mainly affected alanine, aspartate and glutamate metabolism, TCA cycle, glycolysis or gluconeogenesis, butyrate metabolism and pyruvate metabolism, indicating improvements in the urinary metabolism characteristics of depressed rats. Resistance training improved the urine metabolism of depressive rats by regulating the metabolism of alanine, aspartate and glutamate, and TCA cycle. The Kyoto Encyclopedia of Genes and Genomes (KEGG) pathway database (http://www.genome.jp/kegg) was used to retrieve the relevant metabolic pathways and to integrate the metabolic pathways involved in the differential metabolites in the urine of the group D. It can be seen from Fig 8 that CUMS affected energy metabolism (light blue background), amino acid metabolism (light yellow background), and intestinal microbial metabolism (light grey background).

## Discussion

In this study, the CUMS-induced depression model was successfully established. The rats showed typical depressive symptoms such as weight loss, reduction in activity and exploratory behavior, and lack of pleasure (indicated by SPT). After four weeks of aerobic and resistance training, the rate of sucrose preference and the number of crossing and standing were significantly improved, indicating antidepressant effects. However, neither mode of exercise could reverse the weight loss caused by CUMS, possibly because exercise consumed more energy without a significant change in food intake [41, 42].

Urine is the main route for eliminating metabolic waste from the body. The changes of metabolites in urine can reflect not only the characteristics of the body’s overall metabolism, but also abnormal function of tissues or organs. In this study, ^1^H-NMR analysis technique was used, and 21 potential biomarkers were identified in the urine of rats with CUMS-induced depression that were mainly related to energy metabolism, amino acid metabolism and intestinal microbial metabolism. The aerobic and resistance training employed in this study appeared to be able to significantly reverse the effects of depression in five and six biomarkers respectively, which are directly or indirectly related to the metabolic pathways. These changes may have collectively contributed to the improved symptoms of depression by the exercise interventions.

### Energy metabolism

Studies have found that mitochondrial dysfunction or changes in brain energy metabolism may be involved in the pathogenesis of depression [43, 44]. Citrate, α-Oxoglutarate and malate are key intermediates in mitochondrial TCA cycle and all a part of the energy metabolic pathway. In this study, compared with the normal control group, the levels of citrate, α-Oxoglutarate and malate in the rats with depression were significantly decreased, suggesting the effects of the CUMS. Creatine has an important protective effect on mitochondrial energy metabolism, and plays a key role in brain energy homeostasis [45]. A decrease of creatine level is suggested to be related to the pathophysiology of depression [45, 46]. Creatine synthesis is closely related to glucose metabolism. After synthesized by the liver and kidneys, creatine is transported by blood to tissues with high energy requirements, such as the brain and skeletal muscle [47]. The creatine-creatine phosphate-ATP pathway plays a major role in cellular energy transfer. Creatinine is a non-enzymatic catabolic product of creatine and creatine phosphate, which is excreted with urine. Creatine supplements can alter depression-like behavior in rodents [48, 49]. Insufficient energy supply or fatigue is one of the most common symptoms of depression [50, 51]. The results of this study showed that the urine creatine level of the rats with depression was increased and the creatinine level was decreased, suggesting that the creatine-phosphate system was dysfunctional. Leem et al. found that regular exercise combined with creatine supplementation had a greater effect on hippocampal neurogenesis compared with each treatment in chronic mild stress-induced behavioral depression [52]. Interestingly, we found that both aerobic and resistance training could significantly reduce urinary creatine level in depressed rats induced by CUMS. Furthermore, the resistance exercise caused a significant increase of citrate and α-Oxoglutarate levels, indicating that resistance exercise could improve depressive symptoms in rats by regulating energy metabolism pathways such as the TCA cycle. Pyruvate is the end product of glycolysis and is also the starting point for gluconeogenesis. Lower pyruvate levels may indicate that the glycolysis process is impaired. Pyruvate can also be converted to acetyl CoA that enters the TCA cycle. Therefore, a decrease in pyruvate levels can lead to a TCA circulatory disorder. In this study, the pyruvate content in the urine of the rats with depression was reduced, and aerobic training had significantly reversed the pyruvate level. It is also interesting to find that both aerobic and resistance training had common effects on creatine, but different effects on pyruvate, α-Oxoglutarate, and citric acid. In summary, the results suggest that the aerobic and resistance training used in this study improved these biomarkers in the glycolysis and TCA metabolic pathways, in rats with CUMS-induced depression.

### Amino acid metabolism

There has been evidence that abnormalities in the glutamatergic system is associated with the pathophysiology of depression [53–55]. Glutamate is an important excitatory neurotransmitter that plays a vital role in learning and memory. However, increased glutamate levels also induce neurotoxicity [56]. Glutamine is the main precursor of glutamate. Glutamine can circulate in neurons and astrocytes through glutamine-glutamate cycle, so it plays an important role in glutamatergic neurotransmission [57–59]. Several studies have shown that interruption of glutamine-glutamate circulation in the cerebral cortex is closely related to depression-like behavior in animals [60, 61]. In this study, the changes of glutamine could be related to the exposure to CUMS, as the concentration of glutamine in the urine of rats with depression was significantly higher than normal (Table 1). Yang et al. reported that by using LC-MS metabolomics an increase of glutamine concentration was found in the brain of the rats with CUMS-induced depression. The authors suggested that glutamine might be a potential biomarker for the future diagnosis of depression and development of antidepressant [62]. Liu et al. found an increase in plasma glutamine concentration by using ^1^H-NMR metabolomics in patients with depression [63]. It is speculated that the increase in urinary glutamine concentration found in our study might be a compensatory adaptation against glutamate-induced neurotoxicity. In conclusion, both the aerobic and resistance training in this study had significantly down-regulated glutamine levels in the urine, suggesting that the exercise interventions may have a common beneficial effect in regulating the function of the nervous system in depression.

### Intestinal microbial metabolism

Loss of appetite is a common symptom of depression, which is partly associated with abnormal metabolism of the intestinal microbiome. The relationship between gut microbes and brain function has attracted increased attention, and the concept of "gut-brain axis" has emerged [64, 65]. Animal studies have found that altered gut metabolome contributed to depression-like behaviors in rats exposed to CUMS [66]. Clinical studies have shown that patients with depression exhibited a disorder of the gut microbiota, highlighting the potential influence of gut microbiota on the development of depression [67, 68]. In this study, the levels of hippurate, dimethylamine, trimethylamine, and trimethylamine-N-oxide in urine of depressed rats were significantly higher than that of the normal control group, and these metabolites were uniquely produced by bacterial metabolism in the intestinal tract. Therefore, the results suggest that depression-like behavior caused by the CUMS modeling may be related to the changes in intestinal microflora. Yuan et al. found that physical exercise modulated different population of gut bacteria or rich diet, and alleviated gut syndromes to toxin intake. Gut microbiota could as well contribute to the beneficial effects of exercise on cognition and emotion, either directly through serotonin signaling or indirectly by modulating metabolism and exercise performance [69]. Similarly, we found that resistance training significantly reverted the level of trimethylamine-N-oxide, indicating that it has a regulatory effect on the intestinal flora metabolism in depressed rats. However, no significant effects on these metabolites were found after the aerobic training. Further research may explore and confirm why aerobic exercise has a different effect on intestinal microbiome as compared to resistance training.

## Conclusion

This study used ^1^H-NMR-based urine metabolomics for the first time to evaluate the therapeutic effect of aerobic and resistance training on rats with CUMS-induced depression. Twenty-one potential biomarkers in the urine of CUMS rats were identified. Aerobic and resistance training resulted in common effects on the metabolic pathways of alanine-aspartate-glutamate, TCA cycle, and butyric acid. Aerobic training also had effects on glycolysis or gluconeogenesis, and pyruvate metabolism, while resistance training had additional effect on intestinal microbial metabolism. These results provide further insights into the pathophysiology of depression and the mechanisms of the antidepressant effects of aerobic and resistance exercise interventions.
